# Editorial: Machine learning and statistical models: unraveling patterns and enhancing understanding of mental disorders

**DOI:** 10.3389/fpsyt.2025.1588031

**Published:** 2025-05-13

**Authors:** Quan Wang

**Affiliations:** Xi’an Institute of Optics and Precision Mechanics, Chinese Academy of Sciences (CAS), Xi’an, China

**Keywords:** machine learning (ML), artificial intelligence (AI), generative model, mental disorders, explainability

The field of psychiatry has long been grappling with the complexities of mental disorders, which are often characterized by heterogeneous presentations and multifactorial etiologies. Traditional diagnostic methods, largely reliant on clinical interviews and self-reported questionnaires, have limitations in terms of subjectivity and the ability to capture the nuanced biological and psychological underpinnings of these conditions. The advent of machine learning (ML) and advanced statistical models has opened new avenues for unraveling the intricate patterns underlying mental disorders, offering the potential for more accurate diagnosis, prognosis, and personalized treatment strategies. This Research Topic, “Machine Learning and Statistical Models: Unraveling Patterns and Enhancing Understanding of Mental Disorders,” features articles from diverse continents and countries, employing a wide range of methodological approaches, including different levels of statistical analysis and machine learning or deep learning techniques, to showcase the latest advancements in this domain.

In this Research Topic, López Steinmetz et al. present a study utilizing machine learning (ML) models to predict depression emergence in Argentinean college students during COVID-19 quarantine using longitudinal data (N=1492). SVM and logistic regression excelled in classification, while SVR and ridge regression led regression tasks. This study demonstrates the potential of these models to identify at-risk individuals based on psychological inventories, demographic information, and quarantine-related factors. Their results show the ML’s potential for scalable, cost-effective depression screening, offering insights for resource-limited settings. This study highlights the importance of leveraging data-driven approaches to enhance early detection and intervention for depression, particularly in vulnerable populations such as college students.

Another study by Kanyal et al. explores the application of multi-modal deep learning from imaging and genomic data for schizophrenia classification. The authors propose a novel framework that integrates structural MRI, functional MRI, and genetic markers (SNP) to improve diagnostic accuracy. By using explainable AI with layer-wise relevance propagation (LRP) feature selection to identify critical functional connections and SNPs, and fusion of morphological, functional, and genomic features with XGBoost. The model outperforming single-modality approaches, while providing interpretable biomarkers aligned with clinical findings, enhancing both diagnostic precision and biological insight, highlighting the potential of multi-modal approaches to elucidate the complex neurobiological mechanisms underlying this disorder.

Sawalma et al. investigate the utility of data-driven personality measures versus traditional psychological temperaments in psychiatry. By validating an Arabic version of the tri-dimensional personality questionnaire (TPQ) and employing independent component analysis (ICA), the authors construct data-driven personality components that outperform traditional psychological measures in differentiating medication-naïve patients with major depressive disorder from healthy controls. This study emphasizes the importance of re-examining psychometric data through data-driven lenses to improve replicability and clinical utility.

Yamaguchi et al. introduce a generative AI model for simulating structural brain changes in schizophrenia. Using cycle generative adversarial networks (CycleGANs), the authors transform MRI images of healthy individuals into those resembling patients with schizophrenia (SZ), capturing subtle brain volume changes consistent with existing literature. They also simulated disease comorbidities (e.g., ASD + SZ). This innovative approach not only aids in visualizing brain changes associated with schizophrenia but also provides a new tool for simulating the mechanisms of the disorder and to understand the progression.

Yoon et al. present a novel approach to predicting neuroticism using open-ended responses and natural language processing (NLP). It developed a language-based personality assessment model using the five-factor model of personality and the KoBERT pre-trained language model. The study identified effective questions for predicting neuroticism and its facets, such as social comparison and negative feelings. The model’s predictive accuracy was comparable to that of clinical psychology graduate students. This study highlights the potential of NLP in personality assessment and the importance of item content in predicting personality traits, offering practical guidelines for integrating open-ended questions into computational personality research.

Niu et al. developed an explainable predictive model for anxiety risk in Chinese older adults with abdominal obesity using XGBoost and SHapley Additive exPlanations (SHAP) analysis. Leveraging data from 2,427 participants in the CLHLS survey, nine key predictors (e.g., optimism, self-reported health) were identified via LASSO regression. The XGBoost model was interpretable through SHAP, revealing “looking on the bright side” as the most influential feature. With integrating machine learning with SHAP, this work addresses the “black-box” challenge, offering transparent interpretations of the contributing factors, facilitating targeted interventions for high-risk populations.

Lastly, Zhang et al. propose DepITCM, an audio-visual method for detecting depression using multi-task representation learning. By integrating visual and audio features and employing a multi-task learning strategy, the authors achieve significant improvements in depression detection accuracy. This study underscores the potential of multi-modal and multi-task learning approaches in enhancing the robustness and generalizability of mental disorder detection models.

Collectively, these studies exemplify the transformative potential of machine learning and statistical models in psychiatry. They highlight the importance of leveraging diverse data sources, from neuroimaging and genomics to natural language and behavioral data, to uncover the complex patterns underlying mental disorders. Furthermore, they emphasize the need for explainable and interpretable AI models that can bridge the gap between data-driven insights and clinical practice. As we continue to navigate the challenges of mental health in the modern era, the integration of these advanced analytical techniques holds promise for advancing our understanding, improving diagnostic accuracy, and ultimately enhancing patient outcomes.

